# Effect of Body Composition on Supine Pressure Distribution in Bed: A Comparison Between Young and Older Adults

**DOI:** 10.7759/cureus.88987

**Published:** 2025-07-29

**Authors:** Fumiya Ando, Yoshiaki Endo, Yu Terauchi, Ko Onoda

**Affiliations:** 1 Rehabilitation Department, International University of Health and Welfare Shioya Hospital, Yaita, JPN; 2 Department of Physical Therapy, School of Health Sciences, International University of Health and Welfare, Otawara, JPN

**Keywords:** body composition, fat mass, muscle mass, pressure distribution, pressure ulcer prevention

## Abstract

Background: Pressure ulcers are a serious concern in older adults and are often caused by uneven pressure distribution in the supine position. While body mass index is commonly used in risk assessments, it may not reflect localized body pressure. Exploring the relationship between body composition and pressure distribution may help guide individualized prevention strategies.

Objective: To investigate how body composition influences supine pressure distribution across different body regions and to clarify whether these associations differ between healthy young and older adults.

Methods: The mean body pressures in the whole body, upper body, buttocks, and heels were measured using a pressure distribution device (SR Soft Vision, Sumitomo Riko Company Limited, Japan) placed on a pressure-relieving mattress commonly used in clinical settings. Body composition was assessed using a multi-frequency bioelectrical impedance analyzer (InBody S10, InBody Japan Inc., Japan) to obtain body mass index (BMI), skeletal muscle index (SMI), and fat mass index (FMI). Correlation and multiple regression analyses were performed to examine these associations.

Results: Upper-body pressure was significantly higher in older adults, whereas buttock pressure was higher in younger adults. The SMI was significantly associated with body pressure in the younger age group. The FMI was significantly associated with body pressure in the older age group.

Conclusion: Body composition influenced pressure distribution in a region-specific and age-dependent manner. These findings may contribute to the prevention of pressure ulcers and the development of individualized pressure management strategies.

## Introduction

Pressure ulcers are a serious health problem in long-term bedridden patients, particularly older adults, and have been reported to reduce their quality of life significantly [1-3]. Early prevention is essential, as pressure ulcers are progressive and require healing time. Various factors are involved in developing pressure ulcers, of which pressure distribution imbalance is among the most important. Particularly in the back-lying position, weight tends to be concentrated in certain areas, increasing the risk of pressure ulcers in bony prominences such as the buttocks, shoulder blades, and heels [4,5]. Previous studies have shown that in the supine position, body pressure is higher in the sacral and shoulder regions, and that pressure ulcers are more likely to develop in these regions [6]. Thus, the concentration of body pressure in the back-lying position is one of the leading causes of bedsores, especially in older adults. It is thought to become more pronounced owing to changes in support structures associated with aging. Therefore, proper assessment and management of pressure distribution are important to prevent pressure ulcers.

Simple measures, such as body weight and body mass index (BMI), are often used to assess the risk of pressure ulcers [7-11]. However, these indices may not adequately reflect local body pressure on the bony prominences. Previous studies have not found a consistent relationship between BMI and local body pressure. Some studies have reported that a higher BMI tends to increase the pressure in the supine position [12,13], whereas others have found no such relationship [14]. This suggests a more detailed focus on the body components is required to assess the risk of developing pressure ulcers (PUs). The pressure applied to each body part (upper body, buttocks, and heels) may vary greatly depending on the body position, body shape, and muscle/fat distribution. Notably, in the back-sleeper position, the actual risk assessment is limited to a single indicator, due to the different areas where the pressure tends to be localized and concentrated. Several previous studies [14,15] reported that different regions are associated with varying body compositions, with the buttocks suggesting a relationship between body weight and muscle mass. In contrast, no clear correlation was observed in the heel region. In an extensive survey of hospitalized patients [8], the low BMI group reported a significantly higher risk of pressure ulcers in the trunk, including the sacrum, sciatic, greater trochanter, and shoulder areas, suggesting a relationship with trunk BMI. In contrast, no association with BMI was found in the heel region, indicating that the effects of body composition on the risk of pressure ulcers and pressure ulcers differ by region. Other studies [16,17] also reported that decreased muscle mass and malnutrition increase the risk of sacral pressure ulcers, showing a consistent trend. However, studies comparing site-specific associations between younger and older adults and systematically evaluating these associations, including age-related changes, are limited.

Muscle mass tends to decrease with aging [18-20], while fat mass increases [21,22]. These changes decrease body support and pressure-buffering capacity and increase the risk of pressure ulcers. Reduced muscle mass may increase the risk of pressure ulcers in older patients with hip fractures [23]. Thus, a decrease in muscle mass may reduce the cushioning function of subcutaneous tissue and lead to pressure concentration on the bony prominence. However, overweight and obesity have also been reported to increase the risk of pressure ulcers due to greater pressure on the skin and tissues [24]. While fat has a cushioning function, its uneven distribution and sinking may increase local pressure. Changes in muscle and fat mass may affect the body pressure distribution, and the effects of changes in body composition at each site in the supine position remain poorly understood. Elucidating the relationship between aging-related physical changes and body pressure distribution is extremely important for the prevention and rehabilitation planning of pressure ulcers.

This study is novel in that the effects of body composition and age on body pressure distribution in the supine position were analyzed according to measurement sites, such as the upper body, sacral region, and heel region. The study aimed to clarify how body composition affects body pressure distribution in the supine position on the bed. Specifically, we analyzed the relationship between body composition (BMI, skeletal muscle index [SMI], and fat mass index [FMI]) and body pressure at each site (whole-body, upper-body, buttocks, and heels) in healthy young and older participants. We examined the body composition factors affecting body pressure distribution. This study hypothesized that SMI and FMI, among other body compositions, may influence pressure distribution in the back-lying position on the bed. In particular, the effects of muscle mass and fat mass are likely to vary depending on the area of the body under pressure and the age group. In this study, the young and older groups were analyzed independently to explore the differences in the relationship between body composition and body pressure distribution. By comparing younger and older adults, we aimed to clarify the effects of age-related changes in body composition on body pressure distribution in each region and provide fundamental knowledge to better understand the risk of pressure ulcers in the elderly. These findings are expected to contribute to designing individualized interventions for pressure ulcer prevention and management in rehabilitation and nursing care settings.

## Materials and methods

Participants

The study population comprised 729 healthy young adults and 23 healthy older adults. In the older group, 12 participants were men (52%) and 11 were women (48%); in the younger group, 17 participants were men (59%) and 12 were women (41%). The young adult participants were students enrolled at the International University of Health and Welfare who were willing to participate in the study. The healthy older adults were community-dwelling elderly (65 or older) who took part in a health event held in O City and agreed to participate. Convenience sampling was used for participant recruitment, and written informed consent was obtained from all participants. Individuals with a history of orthopedic diseases, limb defects, lower-limb joint disorders affecting range of motion, diseases related to bony prominence, or skin diseases were excluded. The participant characteristics are presented in Table 1.

Measurement of body pressure distribution

**Figure 1:**
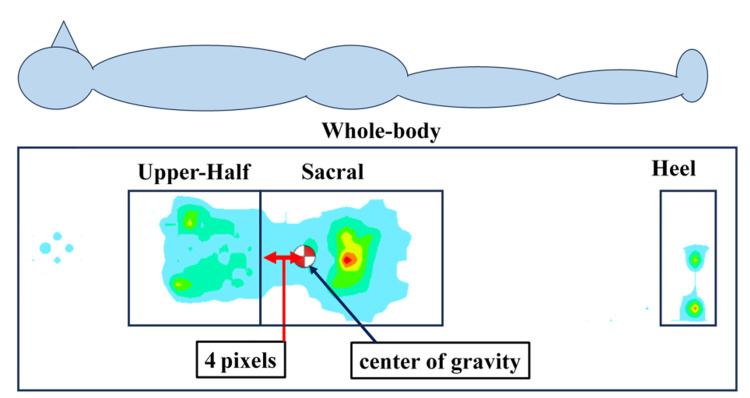
Regions of interest in supine body pressure measurement The image is created by the author. Pressure was measured in four areas: whole body, upper body, sacral region, and heel. Based on anatomical landmarks, the sacral region was defined as the area extending four pixels (approximately 11.2 cm) cephalad from the estimated center of gravity.

The measurements were performed on a nursing bed with three motors (Paramount Bed Co., Ltd., Japan). The beds were covered with an 8 cm-thick pressure-relieving mattress (KE-551; Pregrama Mattress; Paramount Bed Co., Ltd., Japan). Prior to each measurement, the mattress was placed on the sensor sheet without the participant for several seconds, allowing the system to stabilize and adjust baseline pressure values in accordance with the manufacturer's standard procedure. The product consisted of a polyester material with antibacterial, antifungal, and flame-retardant properties, and the filling was 100% polyester. The beds and mattresses used in this study are general-purpose products widely used in medical and nursing care settings in Japan and were employed in this study to reproduce a defined lying environment. Body pressure was measured using a pressure distribution-measuring device (SR Soft Vision, model SVZB922AM; Sumitomo Riko Co., Ltd., Japan). Participants were asked to remain supine and to minimize their body movements during the measurement. The body position of each participant was appropriately adjusted to ensure that the measurement area was within the range of the pressure sensor. The average body pressure values for each selected region were used in the analysis and classified into the following four regions: total body pressure (entire area where pressure can be measured), upper-body pressure (from the measurable area of the neck to the iliac crest), buttock pressure (from the iliac crest to the quantifiable area of the lower buttock), and heel pressure (measurable area of pressure around the heel). The details are presented in Figure 1.

The position of the iliac crest, which separates the measurement range for the upper-body and buttock pressures, was established using the following procedure. According to previous studies, the body's center of gravity is located near the front of the second sacral vertebra [25]. In this study, a physical therapist skilled in palpation measured the distance from the spinous process of the second sacral vertebra to the iliac crest using a measuring tape in five healthy male and five healthy female participants (10 in total). The results showed that the average distance was 9.65 ± 0.75 cm. This study used data displayed in pixels to measure body pressure distribution using SR SoftVision (Sumitomo Riko Company Limited, Japan). Each pixel corresponded to approximately 2.80 cm. Starting from the pixel containing the center of gravity, four consecutive pixels (approximately 11.2 cm) in the direction of the head were defined as the area containing the iliac crest, and the gluteal region was determined based on this area.

Body composition measurement

Body composition was measured using an InBody S10 body composition measuring device (InBody Japan, Inc., Japan). BMI, SMI, and FMI were measured. The BMI, which is calculated as weight (kg) divided by height squared (m²), is widely used as an indicator for an overall assessment of body size, such as obesity and underweight, but has the limitation that it cannot distinguish between muscle mass and fat mass. SMI, calculated as limb skeletal muscle mass (kg) divided by height squared (m²), is widely used to assess sarcopenia due to aging and disease. FMI is calculated as the amount of body fat (kg) divided by the square of the height (m²). Unlike BMI, FMI can explicitly evaluate fat mass and is a valid objective indicator of body fat excess or deficiency. In accordance with general BIA guidelines, all measurements were performed while avoiding the immediate postprandial period to minimize the influence of recent food and fluid intake on body composition values. 

Statistical analysis

This study examined the differences and associations between body composition and pressure distribution in healthy young and elderly individuals. First, an unpaired t-test (or Mann-Whitney U test, depending on the normality of the variables) was conducted to determine whether there were differences in body composition (BMI, SMI, and FMI) and body pressure (whole-body, upper-body, buttocks, and heel) between healthy young and older groups. This revealed the underlying differences between the age groups.

A two-stage analysis explored the relationship between the older group's body composition and body pressure distribution. The relationship between body composition indices (BMI, SMI, and FMI) and body pressure (whole-body, upper-body, buttocks, and heel) was examined using Pearson's correlation analysis; Spearman's rank correlation was used when normality was not met. A multiple regression analysis was conducted using only the body composition indices that showed significant associations in the correlation analysis as explanatory variables, with each body pressure site as the dependent variable. In all models, sex was forced into Block 1 as a covariate, and the explanatory variable (body composition) was selected using a stepwise method in Block 2. This allowed us to evaluate the independent effects of body composition on body pressure after adjusting for sex. Separate models were constructed for each type of body pressure (whole-body, upper-body, buttocks, and heels). To eliminate the influence of multicollinearity among the explanatory variables in the multiple regression analysis, each variable's variance expansion factor (VIF) was calculated, and the analysis was conducted after confirming that the VIF was < 10.

All statistical analyses were performed using IBM Corp. Released 2019. IBM SPSS Statistics for Windows, Version 28.0. Armonk, NY: IBM Corp. The significance level was set at p < 0.05.

Ethical considerations

This study was conducted following the Declaration of Helsinki and was approved by the Research Ethics Review Committee at the International University of Health and Welfare (approval no. 23-Io-47) on December 20, 2023. The purpose and content of the study were explained to the participants in advance, and their consent was obtained before measurements began. Minors' permission was obtained from their parents or guardians.

Data collection was conducted from late December 2023 to December 2024.

## Results

Associations between the body composition indices (BMI, SMI, and FMI) and pressure distribution were analyzed for each age group and body region (Table 1).

**Table 1:** Comparison of pressure distribution and body composition between older and younger adults Values are presented as mean ± standard deviation. Depending on normality, p-values were calculated using independent t-tests or Mann-Whitney U-tests. The Mann-Whitney U-test was used for heel pressure due to non-normal distribution. Cohen’s d effect sizes were interpreted as small (0.2), medium (0.5), and large (0.8). All pressure values were measured in millimeters of mercury (mmHg). BMI: body mass index; SMI: skeletal muscle mass index; FMI: fat mass index

	Older Adults (n = 23)	Younger Adults (n = 29)	p-value	Cohen’s d
Whole-body pressure, mmHg	30.3 ± 3.1	30.2 ± 2.7	0.883	0.04
Upper-body pressure, mmHg	32.0 ± 5.4	28.3 ± 3.9	< 0.001	0.81
Sacral pressure, mmHg	33.2 ± 4.4	35.6 ± 4.2	0.049	-0.56
Heel pressure, mmHg	31.7 ± 7.7	34.9 ± 8.5	0.167	-0.39
Age, years	72.6 ± 4.8	20.1 ± 1.2	< 0.001	15.9
BMI, kg/m²	24.5 ± 2.3	22.0 ± 2.1	< 0.001	1.18
SMI, kg/m²	7.0 ± 0.9	7.2 ± 1.1	0.553	-0.17
FMI, kg/m²	7.0 ± 1.4	4.5 ± 1.9	< 0.001	1.45

Comparison of basic attributes and body composition

Significant differences (p < 0.001) were found between younger and older adults regarding age, BMI, and FMI, all of which were higher in the older adults. In contrast, there was no significant difference in SMI (p = 0.553). The results are summarized in Table 1.

Comparison of body pressure distribution

**Figure 2:**
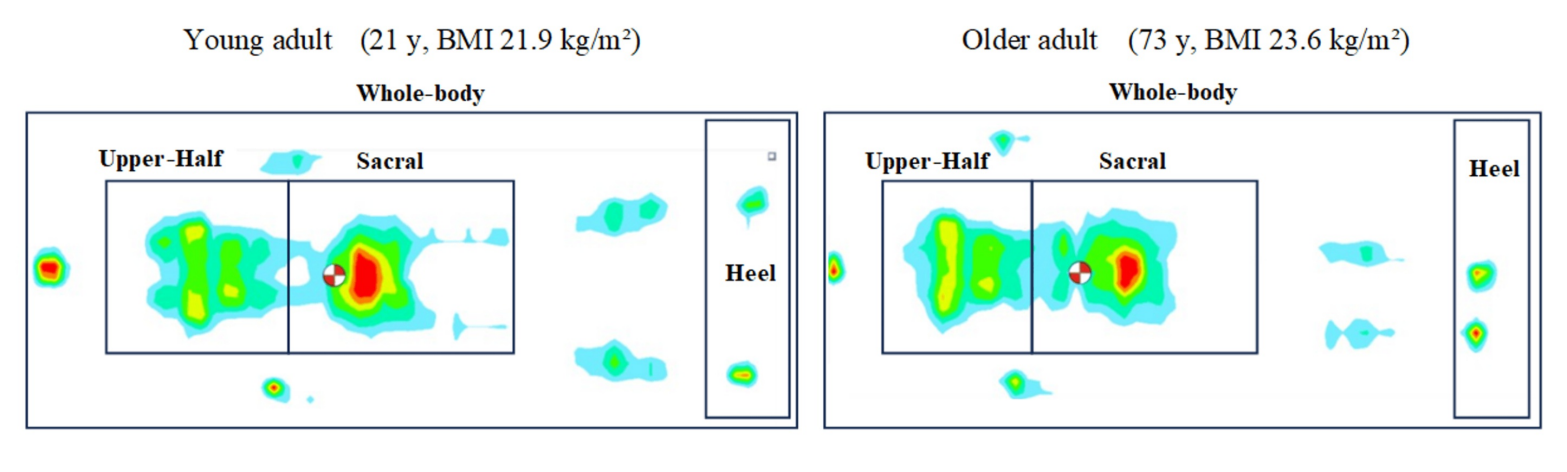
Representative supine pressure-distribution heatmaps (young vs. older adults) To illustrate typical pressure distribution patterns, we selected male participants from each age group whose BMI was closest to the group median and whose pressure maps visually represented characteristic distribution features. The young adult was 21 years old with a BMI of 21.9 kg/m², and the older adult was 73 years old with a BMI of 23.6 kg/m². A shared color scale is applied. Panel A highlights a higher-pressure zone over the sacral area, whereas panel B shows a shift toward the upper-torso region. These patterns visually reinforce the statistically significant age-related differences reported in the study (greater sacral loading in younger participants vs. greater upper-body loading in older participants).

Upper-body pressure was significantly higher in older patients (p = 0.006, d = 4.63), and sacral region pressure was significantly higher in younger patients (p = 0.049, d = 4.32). No significant differences were found in mean total body pressure (p = 0.883) or heel pressure (p = 0.167). The results are summarized in Table 1. To help visualize these age-specific patterns, Figure 2 presents representative supine pressure heatmaps from young and older participants whose overall pressure distributions are close to the group medians.

Relationship between body pressure and body composition at each site

The relationships between body composition (BMI, SMI, FMI) and regional body pressure were analyzed for younger and older adults separately using correlation and multiple-regression analyses. Detailed results are presented in Tables 2-5.

**Table 2:** Correlation coefficients between body composition variables and pressure distribution in younger adults Values are Pearson’s r (p-value) unless otherwise indicated. Statistical significance was set at p < 0.05. Pearson’s correlation coefficients are presented; Spearman’s rank correlation was applied for heel pressure owing to non-normal distribution. Significant correlations are shown in bold. Mean ± standard deviation (SD) values are shown in parentheses next to variable names. BMI: body mass index; SMI: skeletal muscle mass index; FMI: fat mass index.

Measurement Site	BMI (22.0 ± 2.1 kg/m²)	SMI (7.2 ± 1.1 kg/m²)	FMI (4.5 ± 1.9 kg/m²)	Age (20.1 ± 1.2 years)
Whole-body pressure (30.2 ± 2.7 mmHg)	0.096 (p = 0.620)	0.596 (p = 0.001)	–0.459 (p = 0.012)	0.029 (p = 0.883)
Upper-body pressure (28.3 ± 3.9 mmHg)	0.326 (p = 0.084)	0.488 (p = 0.007)	–0.208 (p = 0.279)	0.116 (p = 0.547)
Sacral pressure (35.6 ± 4.2 mmHg)	–0.090 (p = 0.641)	0.431 (p = 0.020)	–0.421 (p = 0.023)	–0.087 (p = 0.655)
Heel pressure (34.9 ± 8.5 mmHg)	0.074 (p = 0.701)	0.082 (p = 0.672)	0.033 (p = 0.864)	0.046 (p = 0 .813)

**Table 3:** Correlation coefficients between body composition variables and pressure distribution in older adults Values are Pearson’s r (p-value) unless otherwise indicated. Statistical significance was set at p < 0.05. Pearson’s correlation coefficients are presented; Spearman’s rank correlation was applied for heel pressure owing to non-normal distribution. Significant correlations are shown in bold. Mean ± standard deviation (SD) values are shown in parentheses next to variable names. BMI: body mass index; SMI: skeletal muscle mass index; FMI: fat mass index.

Measurement Site	BMI (24.5 ± 2.3 kg/m²)	SMI (7.0 ± 0.9 kg/m²)	FMI (7.0 ± 1.4 kg/m²)	Age (72.6 ± 4.8 years)
Whole-body pressure（30.3 ± 3.1 mmHg）	0.283 (p = 0.191)	0.006 (p = 0.979)	0.456 (p = 0.029)	0.207 (p = 0.344)
Upper-body pressure（32.0 ± 5.4 mmHg）	0.283 (p = 0.191)	0.378 (p = 0.075)	0.143 (p = 0.515)	0.414 (p = 0.049)
Sacral pressure（33.2 ± 4.4 mmHg）	0.288 (p = 0.183)	-0.203 (p = 0.353)	0.597 (p = 0.003)	0.001 (p = 0.999)
Heel pressure（31.7 ± 7.7 mmHg）	-0.252 (p = 0.245)	-0.231 (p = 0.288)	-0.027 (p = 0.903)	0.267 (p = 0.219)

**Table 4:** Associations between body composition and pressure distribution in young adults Values are Pearson’s r (p-value) for correlations and standardized β (p-value) for multiple-regression analyses. Statistical significance was set at p < 0.05. Statistically significant values are shown in bold. Pearson’s correlation coefficients are presented; Spearman’s rank correlation was applied for heel pressure owing to non-normal distribution. Sex was forced into the model as a covariate; SMI and FMI were entered using the stepwise method. Only variables that showed significant bivariate correlations were retained in the final models. Power values were obtained via post-hoc GPower analysis (α = 0.05; fixed model, R² deviation from zero). Values ≥ 0.80 are considered adequate. Mean ± SD values for the variables in young adults were as follows: BMI = 22.0 ± 2.1 kg/m², SMI = 7.2 ± 1.1 kg/m², FMI = 4.5 ± 1.9 kg/m², Age = 20.1 ± 1.2 years, 17 males (59%); 12 females (41%).

Region	Significant Correlations	Significant Predictors (β, p)	Adjusted R²	Post-hoc Power (1−β
Whole-body pressure (30.2 ± 2.7 mmHg)	SMI：r = 0.596, p = 0.001	Sex (β = –0.598, p = 0.001)	0.334	0.97
	FMI：r = –0.459, p = 0.012	SMI (β = 0.311, p = 0.325) – excluded		
		FMI (β = –0.162, p = 0.407) – excluded		
Upper-body pressure (28.3 ± 3.9 mmHg,)	SMI：r = 0.488, p = 0.007	Sex (β = 0.423, p = 0.217) – excluded	0.227	0.82
		SMI (β = 0.855, p = 0.017)		
Sacral pressure (35.6 ± 4.2 mmHg)	SMI：r = 0.431, p = 0.020	Sex (β = –0.479, p = 0.009)	0.200	0.80
	FMI：r = –0.421, p = 0.023	SMI (β = 0.494, p = 0.551) – excluded		
		FMI (β = –0.195, p = 0.652) – excluded		
Heel pressure (34.9 ± 8.5 mmHg)	No significant correlations	Not analyzed	—	—

**Table 5:** Associations between body composition and pressure distribution in older adults Values are Pearson’s r (p-value) for correlations and standardized β (p-value) for multiple-regression analyses. Statistical significance was set at p < 0.05. Statistically significant values are shown in bold. Pearson’s correlation coefficients are presented; Spearman’s rank correlation was applied for heel pressure owing to non-normal distribution. Sex was forced into the model as a covariate; SMI and FMI were entered using the stepwise method. Only variables that showed significant bivariate correlations were retained in the final models. Power values were obtained via post-hoc GPower analysis (α = 0.05; fixed model, R² deviation from zero). Values ≥ 0.80 are considered adequate. Mean ± SD values for the variables in older adults were as follows: BMI = 24.5 ± 2.3 kg/m², SMI =7.0 ± 0.9 kg/m², FMI =7.0 ± 1.4 kg/m², Age = 72.6 ± 4.8 years, 12 males (52%); 11 females (48%). SMI: skeletal muscle mass index; FMI: fat mass index; R²: adjusted coefficient of determination.

Region	Significant Correlations	Significant Predictors (β, p)	Adjusted R²	Post-hoc Power (1−β
Whole-body pressure（30.3 ± 3.1 mmHg）	FMI：r = 0.456, p = 0.029	Sex (β = 0.184 p = .401) – excluded	0.178	0.53
		FMI (β =0.439, p = 0.046)		
Upper-body pressure（32.0 ± 5.4 mmHg）	Age: r = 0.414, p = 0.049	Sex (β = –0.575, p = 0.087) – excluded	—	0.43
		Age (β = –0.304, p = 0.258) – excluded		
Sacral pressure（33.2 ± 4.4 mmHg）	FMI：r = 0.597, p = 0.003	Sex (β = 0.509, p = 0.013)	0.433	0.98
		FMI (β = 0.494, p = 0.008)		
Heel pressure（31.7 ± 7.7 mmHg）	No significant correlations	Not analyzed	—	—

In the younger group, SMI showed positive correlations with whole-body, upper-body, and sacral pressures, whereas FMI correlated negatively with whole-body and sacral pressures. Multiple-regression analysis identified SMI as an independent predictor of upper-body pressure, and sex emerged as a significant covariate for whole-body and sacral pressures.

In the older group, FMI was positively correlated with whole-body and sacral pressures and served as the only significant explanatory variable for these two regions in the regression models. Upper-body pressure correlated positively with age, but age did not remain significant in the regression model.

No significant associations were found for heel pressure in either age group, and therefore, regression analysis was not performed for this region.

## Discussion

This study examined the relationship between body pressure distribution and body composition in young and elderly individuals. Body pressure distribution showed a trend toward higher pressure in the sacral region in younger patients and higher upper-body pressure in older patients. Body composition was mainly associated with muscle mass (SMI) in the younger group and fat mass (FMI) in the older group. These results suggest that age-related body structure and function changes may affect pressure distribution and background factors differently.

Younger participants tended to have higher body pressures in the sacral region, whereas older participants had higher upper-body pressures. These differences in body pressure distribution may be due to age-related differences in the postural characteristics. Older adults, such as those with pelvic retroversion and kyphosis, are particularly prone to age-related postural changes [20]. This increases the contact area of the upper body and may be a structural factor that leads to increased upper-body pressure. In contrast, younger patients have higher postural retention and better skeletal alignment. Therefore, it is presumed that in the supine position, loads tend to be concentrated around the pelvis, and the pressure on the sacral region is relatively high. Aging changes in the skin and subcutaneous tissues may also affect body pressure distribution. In older adults, the epidermis thins, the bonds between the stratum corneum cells weaken, and the dermis becomes less elastic due to the degeneration and reduction of collagen and elastin fibers [21]. Furthermore, subcutaneous tissue decreases with age and may have a reduced role as a buffer [22]. Although posture and subcutaneous tissue structure were not directly measured in this study, the results suggest that posture- and age-related changes in the skin and subcutaneous tissue and body composition may be the background factors affecting body pressure distribution.

In this study, the relationship between body pressure distribution and body composition was examined by age group and found to be mainly related to muscle mass (SMI) in the younger group and fat mass (FMI) in the older group. This study found a significant positive correlation between the upper-body pressure and SMI in young individuals; SMI was the only significant explanatory variable in the multiple regression analysis. These results suggest that muscle mass has a pronounced effect on body pressure distribution in young adults. Higher upper-body pressure in young people can be attributed to the structural characteristics of the upper body. The upper body generally has a high concentration of heavy structures, such as the rib cage and internal organs, and is a relatively large part of the entire body. Previous research showed that in men, the combined upper-body weight of the chest and abdomen accounts for approximately 40% or more of the total body weight and is greater than the total weight of the lower extremities (thighs, lower legs, and feet combined) [23]. In addition, young adults have a high SMI and retain muscle mass in the trunk, which further increases upper-body mass owing to the weight of the muscles themselves [24,25]. This may have led to an increase in the upper-body pressure. In the same group of young adults, correlations were found among total body pressure, sacral body pressure, and body composition. The multiple regression analysis showed a statistically significant relationship with upper-body pressure. This suggests that the relationship between body pressure distribution and muscle mass may differ by region and be particularly pronounced in the upper body.

In the elderly, significant positive correlations were found among total body pressure, sacral body pressure, and FMI, with FMI being the only significant explanatory variable extracted from the multiple regression analysis. These results suggest that higher fat mass in older adults results in higher body pressure, and age-related changes in fat may affect the increase in pressure. A previous study [16] reported a trend toward an increase in abdominal and perivisceral fat and a decrease in subcutaneous fat in the lower extremities with age. Furthermore, intramuscular and visceral fat accumulation progresses with age, while subcutaneous fat in the extremities relatively decreases [15]. The function of enzymes involved in estrogen production in the adipose tissue of the buttocks and abdomen increases with age, indicating that the metabolic role of adipose tissue changes with age [26]. These changes make it easier for fat to concentrate in the trunk region of older adults. This structure is prone to high body pressure, particularly in the sacral region and other support surfaces. In addition, aging, which reduces the elasticity of subcutaneous fat and its cushioning function, may interfere with pressure dispersion and contribute to a localized pressure increase [21,22]. Increasing and redistributing body fat in older adults are essential factors that directly influence pressure distribution. Future pressure management strategies for preventing pressure ulcers should consider the location and properties of fat accumulation.

The results showed that muscle mass (SMI) and fat mass (FMI) affected different sites in younger and older individuals, respectively, providing a practical perspective on pressure ulcer prevention and pressure management and clearly showing how changes in body structure and composition with aging affect body pressure distribution by site. Although BMI and body weight have traditionally been used for pressure ulcer prevention [8,10], these systemic indicators alone may not be sufficient to assess the risk of pressure distribution. This study provides a new perspective for future pressure ulcer risk assessments by examining the relationship between more detailed body composition assessments (SMI and FMI) and pressure distribution. In conclusion, this study is clinically significant, as it provides new findings that contribute to our understanding of body pressure distribution by considering differences in age and body composition and specific suggestions for site-specific pressure management.

In the present study, no significant associations were observed between heel pressure and BMI, SMI, or FMI in either age group. However, this result warrants cautious interpretation because the heel is one of the most common sites of pressure ulcer development [4,5]. Heel loading is likely influenced more by local anatomical conditions, particularly the thickness and viscoelasticity of the heel fat pad, than by whole-body body composition indices. A large-scale ultrasound study involving 1,126 Japanese participants reported that heel fat pad thickness peaks between ages 30 and 44 and gradually declines with age [27]. Furthermore, reductions in heel fat pad thickness have been associated with plantar heel pain [28], suggesting that the quality of local soft tissues may play a more critical role in heel-related pressure pathology than systemic metrics such as BMI or FMI. Additionally, due to the small contact area of the heel, even slight plantarflexion or dorsiflexion of the ankle can significantly alter the measured pressure values, potentially masking subtle body composition-related effects. Future studies should consider combining localized soft tissue evaluations (e.g., ultrasound-based heel fat pad assessment) with high-resolution or dynamic pressure mapping to better understand the biomechanical underpinnings of heel pressure and its contribution to pressure ulcer risk.

This study had several limitations. First, the study was limited to healthy young and older adults and did not include individuals with conditions known to place them at high risk for pressure ulcers. Patients with chronic diseases such as diabetes, hypertension, asthma, and chronic obstructive pulmonary disease are reported to have a significantly higher risk of developing pressure ulcers [29,30]. For example, it has been reported that diabetic patients have an approximately 1.77-fold increased risk of pressure ulcers compared to non-diabetic patients [31]. Against this background, future studies on body pressure distributions in disease groups may provide more clinically meaningful findings. Second, the evaluation of body pressure distribution was limited to static measurements simultaneously and did not include dynamic evaluations, such as pressure changes with positional changes or over time. Given that pressure is expected to fluctuate over time under actual lying conditions, introducing a study design that can track changes over time is recommended. In addition, due to the limited number of participants in this study, the statistical power was insufficient for a detailed examination of the effects of sex and body size. In the future, the number of subjects should be increased. Third, post-hoc power analysis showed that while several regression models (e.g., whole-body and sacral pressure in young adults) achieved adequate power (≥ 0.80), others, particularly the upper-body and whole-body models in older adults, exhibited low power (< 0.60), indicating a considerable risk of Type II error and underscoring the need for larger, more balanced samples based on a priori power calculations. A more detailed analysis of sex, age, and body size would allow for a deeper understanding of the individual characteristics of body-pressure distribution and its related factors. Moreover, the use of multiple-regression analysis with a relatively small sample size may introduce potential bias and overfitting. Although we attempted to mitigate this by limiting the number of explanatory variables through stepwise selection and confirming variance inflation factors, the results should be interpreted with caution. Future studies with larger cohorts are warranted to confirm the robustness of the associations found in this study. Fourth, while we examined the associations between SMI and FMI and regional body pressure distribution separately, we did not explore composite risk indicators that integrate these components (e.g., regional SMI/FMI ratios reflecting muscle-fat balance). Future studies incorporating such composite indices may enhance the predictive accuracy of pressure ulcer risk and support their use as clinical screening tools. Fifth, while our study focused on regional pressure distribution and its relationship with body composition, we did not assess functional skin outcomes such as skin integrity scores or early signs of pressure injury. Future research integrating such clinical indicators could bridge pressure mapping with actual patient outcomes and enhance its clinical relevance. Finally, several potential confounding factors related to measurement conditions should also be acknowledged. For example, hydration status is known to affect the accuracy of bioelectrical impedance analysis (BIA). Habitual fluid intake, the timing and amount of water consumed on the day of measurement, and the time elapsed since last intake may have influenced body composition readings [32]. Standardizing pre-measurement hydration protocols may improve consistency. Additionally, habitual sleeping posture could affect resting muscle tone and soft tissue distribution [33], which may influence pressure mapping. Regarding support surfaces, although a standard hospital-type mattress was used for all participants, differences in mattress compliance, such as the use of pressure-relief mattresses in clinical settings, could alter body pressure distributions. These factors should be considered in future research designs and when applying results to clinical practice.

## Conclusions

In conclusion, this study revealed that the body pressure distribution in the supine position was differentially associated with age and body composition, particularly SMI and FMI, on a site-by-site basis. SMI and FMI significantly affected body pressure in the younger and older participants, respectively, suggesting the importance of capturing age-related changes in body composition on body pressure distribution by site. The findings of this study are expected to provide basic data for pressure ulcer prevention and the development of individualized pressure management strategies and contribute to the advancement of assessment and intervention methods in rehabilitation and long-term care settings in the future.
